# *QuickStats*: Percentage* of Adults Aged ≥18 Years Who Currently Use E-Cigarettes,^†^ by Sex and Age Group — National Health Interview Survey,^§^ 2016

**DOI:** 10.15585/mmwr.mm665152a7

**Published:** 2018-01-05

**Figure:**
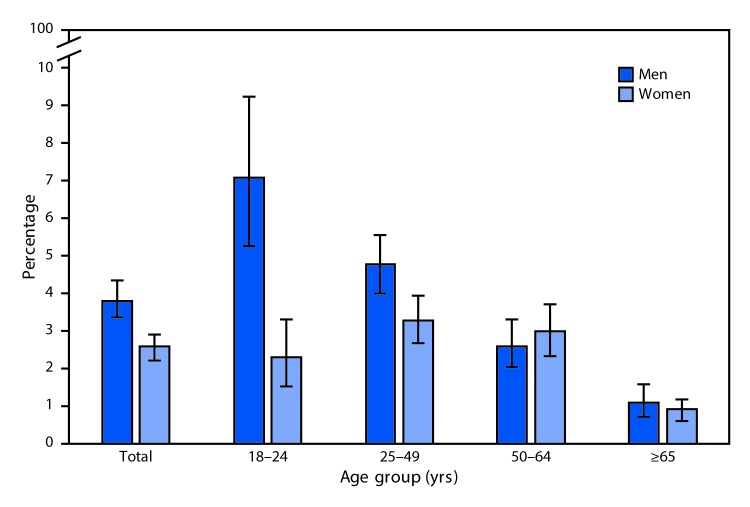
In 2016, 3.8% of men and 2.6% of women aged ≥18 years currently used e-cigarettes. Among men, current e-cigarette use decreased with advancing age, from 7.1% among men aged 18–24 years to 4.8% among men aged 25–49 years, 2.6% among men 50–64 years, and 1.1% among men aged ≥65 years. Among women, current e-cigarette use increased between ages 18–24 years (2.3%) and 25–49 years (3.3%) and decreased between ages 50–64 years (3.0%) and ≥65 years (0.9%). A greater percentage of men aged 18–24 years and 25–49 years currently used e-cigarettes compared with women in the same age groups.

**Source:** National Health Interview Survey, 2016. https://www.cdc.gov/nchs/nhis/index.htm.

